# Scaling transformers to high-dimensional sparse data: a Reformer-BERT approach for large-scale classification

**DOI:** 10.3389/frai.2025.1661318

**Published:** 2025-11-17

**Authors:** Wanxuan Li, Xinhua Li, Weihang Guo, Boyuan Gu, Jianjun Du, Ning Chi, Dan Shao, Kai Xiao, Ren Mo

**Affiliations:** 1 Department of Urology, Inner Mongolia People’s Hospital, Inner Mongolia Urological Institute, Hohhot, China; 2 School of Medicine, South China University of Technology, Guangzhou, China; 3 Affiliated Inner Mongolia Clinical College of Inner Mongolia Medical University, Hohhot, China; 4 Institutes of Biomedical Sciences, Inner Mongolia University, Hohhot, China

**Keywords:** cell type, major cell categories, classification, gene expression, scRNA-seq

## Abstract

**Objective:**

The precise identification of human cell types and their intricate interactions is of fundamental importance in biological research. Confronted with the challenges inherent in manual cell type annotation from the high-dimensional molecular feature data generated by single-cell RNA sequencing (scRNA-seq)—a technology that has otherwise opened new avenues for such explorations—this study aimed to develop and evaluate a robust, large-scale pre-trained model designed for automated cell type classification, with a focus on major cell categories in this initial study.

**Methods:**

A novel methodology for cell type classification, named scReformer-BERT, was developed, leveraging a BERT (Bidirectional Encoder Representations from Transformers) architecture that integrates Reformer encoders. This framework was subjected to extensive self-supervised pre-training on substantial scRNA-seq datasets, after which supervised fine-tuning and rigorous five-fold cross-validation was performed to optimize the model for predictive accuracy on targeted first-tier cell type classification tasks. A comprehensive ablation study was also conducted to dissect the contributions of each architectural component, and SHAP (SHapley Additive exPlanations) analysis was used to interpret the model’s decisions.

**Results:**

The performance of the proposed model was rigorously evaluated through a series of experiments. These evaluations, conducted on scRNA-seq data, consistently revealed the superior efficacy of our approach in accurately classifying major cell categories when compared against several established baseline methods and the inherent difficulties in the field.

**Conclusion:**

Considering these outcomes, the developed large-scale pre-trained model, which synergizes Reformer encoders with a BERT architecture, presents a potent, effective and interpretable solution for automated cell type classification derived from scRNA-seq data. Its notable performance suggests considerable utility in improving both the efficiency and precision of cellular identification in high-throughput genomic investigations.

## 1 Introduction

Accurate identification and quantification of cells hold significant potential in advancing biological research (Hooke, 1665). Single-cell RNA sequencing (scRNA-seq) has emerged as a powerful tool for exploring cell biology and understanding disease mechanisms. One of the most crucial tasks in scRNA-seq analysis is the precise identification of different cell types. This is crucial for understanding complex biological systems and serves as a foundational prerequisite for numerous advanced downstream applications, from deciphering cellular heterogeneity in cancer (Sefer, 2025) to predicting cell-specific drug responses (Eralp and Sefer, 2024) and informing the design of targeted therapies like RNA nanomedicine (Dwivedi et al., 2025). These cell types are often organized hierarchically, from broad categories to more specific subtypes. After the cell types identified, scRNA-seq could provide valuable insights into the differences in cellular functions across different tissues, as well as the changes in cell types during various stages of differentiation within the same tissue. It also could uncover distinctions between cell types within homogeneous tissues. Despite its promising potential, the annotation of cell data in scRNA-seq is still largely a manual process. This is primarily due to the inherent noise in single-cell sequencing technology and the complexity of handling high-dimensional molecular data.

Machine learning offers a more efficient and convenient approach to processing highly complex scRNA-seq data. Unsupervised clustering is currently one of the most widely used machine learning methods for cell annotation. For instance, RacelD employs K-means clustering to identify different cell subtypes (Grün et al., 2015). ScDAE utilizes a multi-layer denoising autoencoder to build a deep neural network (DNN) model that combines single-cell subtype classification with feature extraction (Choi et al., 2021). SNN-clip groups cells of the same subtype by constructing a shared K-nearest neighbor (KNN) graph, augmented by the SNN similarity measure (Xu and Su, 2015). The growing accumulation of scRNA-seq data has recently led to the increased use of supervised algorithms. For example, Scpred applies singular value decomposition for data compression and then uses a support vector machine (SVM) model for classification (Alquicira-Hernandez et al., 2019). ItClust, on the other hand, integrates target data with transfer learning to classify cell types (Hu et al., 2020). Lin et al. (2017) proposed a neural network-based dimensionality reduction method with supervision (Van der Maaten and Hinton, 2008). Supervised algorithms leverage the rich information in labeled scRNA-seq datasets to build models that can predict and classify unlabeled scRNA-seq data. Both unsupervised and supervised approaches have their respective advantages and limitations. The unsupervised approach does not require large amounts of labeled scRNA-seq data and can characterize cell types and states through clustering. However, increasing cell numbers and batch effects pose significant computational challenges. In contrast, the supervised approach heavily relies on labeled data, which can limit its ability to accurately classify cell types that are specific to the target data but not present in the source data, leading to poor generalization.

Large-scale pre-training models based on Transformer (Vaswani et al., 2023) variants have emerged as a successful global paradigm, particularly in the areas of image processing and natural language processing, according to studies currently performed. Transformer is a foundational architecture behind large language models, designed to process sequential data by employing self-attention mechanisms. It allows the model to weigh the importance of different elements, facilitating efficient processing and understanding of complex patterns. Since the collection of unlabeled data is simpler, supervised fine-tuning for representation learning of large-scale unlabeled data is a breakthrough in both effect and cost. Recently, scBERT adopts the architecture of large-scale pre-trained language model Bert (Devlin et al., 2019), creating the application of Transformer in scRNA sequence data analysis and verifying the self-supervision of pre-training and fine-tuning Paradigm’s ability to learn from unlabeled scRNA-seq data (Yang et al., 2022). While scBERT demonstrated the utility of Transformers, its standard architecture can be computationally intensive for the full spectrum of genes, often necessitating gene filtering. Our work builds upon this by integrating Reformer, aiming to enhance efficiency with long sequences without sacrificing comprehensive gene input. BERT uses the encoder part of the Transformer for feature extraction to perform large-scale pre-training. Furthermore, accurate and scalable cell annotation is a prerequisite for advanced applications, from tracking developmental trajectories in cancer (Eralp and Sefer, 2024) and predicting drug responses (Sefer, 2025) to informing the design of targeted therapies like RNA nanomedicine (Dwivedi et al., 2025). In this paper, we use a large, publicly available heart cell dataset to fine-tune and evaluate a computational method for a first-tier cell type classification model based on the large-scale pre-training model Reformer (Kitaev et al., 2020), to identify the major cell categories of heart cells. Reformer is an efficient variant of the Transformer architecture that addresses the computational limitations of traditional Transformer models when processing long sequences. Unlike standard Transformers that have quadratic complexity with respect to sequence length, Reformer achieves logarithmic complexity through two key innovations: (1) replacing the traditional attention mechanism with locality-sensitive hashing (LSH) attention, which reduces the need to compare each position with all others, and (2) using reversible residual layers that allow for more memory-efficient backpropagation. Based on BERT’s architecture, we use Reformer as an encoder, which preserves the complete gene interpretation, and our model does not depend on the sensitivity of hyperparameters, which makes our method robust. By leveraging Reformer’s efficient attention mechanism, our approach can handle the full set of over 10,000 genes per cell without requiring feature selection or dimensionality reduction techniques that might discard important biological information. This comprehensive approach enables more accurate and potentially more biologically meaningful cell type classification while maintaining computational feasibility.

## 2 Materials and methods

### 2.1 Dataset

Prior to fine-tuning on the specific heart dataset, scReformer-BERT underwent extensive self-supervised pre-training on a large compendium of publicly available, unlabeled scRNA-seq datasets, aggregating ~15 million cells from sources such as the Human Cell Atlas, Tabula Sapiens, and cellxgene (Regev et al., 2017; The Tabula Sapiens Consortium et al., 2022; Megill et al., 2021; The Tabula Muris Consortium, 2018; Chen et al., 2021; Zeng et al., 2021; Uhlén et al., 2015). The heart cell dataset used for fine-tuning and evaluation in this paper is sourced from the 2022 Digital China Innovation Competition, specifically the Digital Medical Track Algorithm Contest. The data is an aggregation from various public datasets (e.g., Tucker et al., 2020), resulting in a substantial volume of information specifically pertaining to cardiac cells. The dataset was generated using the “10X Genomics” sequencing technology (Zheng et al., 2017), a high-throughput platform widely used in single-cell RNA sequencing, which delivers high-quality single-cell transcriptomic data. Among them, the features are represented as a gene expression (cell × gene) matrix. The labels file contains labels for each cell across the four levels of the uHAF tree (a cell may have at most one label per level). This study focuses on the classification of the nine major cell categories at the first tier of this uHAF tree. If some leaf nodes of the uHAF tree do not extend to the fourth layer, the last few layers’ labels of the relevant cells in the label file correspond to the branch’s leaf nodes. Conversely, if some cells cannot be assigned to leaf nodes and can only be designated to an internal node, the subsequent layers of labels will be consistent with the deepest assignable internal node.

### 2.2 The challenge: scaling transformers to high-dimensional transcriptomes

Transformer is an encoder-decoder structure based on global modeling, which can achieve better results on many tasks today. Although the self-attention mechanism is extremely effective, the memory and computing power it requires will grow flat with the length of the sequence. Therefore, the input length of the Transformer is usually limited to no more than 512, and most of our scRNA-seq the case where the data contains more than 10,000 genes makes adopting Transformer-like models a challenge. In recent years, researchers have improved and created Transformer models that can input long sequences in response to the shortcomings of Transformer, which has a large amount of calculation and takes up a lot of memory. CosFormer focuses on the non-negativity of the matrix and achieves an attention mechanism comparable to or even better in long texts by amplifying the local attention weight value (Qin et al., 2022). Transformer-XL divides the long sequence into small segments with a length of 512 and then uses attention across sequences for joint feature modeling (Dai et al., 2019). Longformer adopts an attention pattern to sparse the complete attention matrix, thus enhancing the ability to process long sequences (Beltagy et al., 2020). Performer linearizes the complexity of standard attention through random projection (Choromanski et al., 2021). Linformer proposed a low-rank approximation to implement a new self-attention mechanism to reduce time and space complexity (Wang S. et al., 2020). Reformer’s most notable innovation is the introduction of an attention mechanism grounded in a locally sensitive hashing algorithm, combined with the utilization of reversible residual connections instead of conventional residual connections. This approach effectively reduces both the number of parameters and memory usage.

### 2.3 Model construction

Our model, scReformer-BERT, adapts the Bidirectional Encoder Representations from Transformers (BERT) framework by replacing the standard Transformer encoder with the highly efficient Reformer encoder. This core modification enables us to process the entire, unfiltered gene space of each cell. The overall workflow follows the established two-stage paradigm: a comprehensive self-supervised pre-training phase on a large corpus of unlabeled cells, followed by a supervised fine-tuning phase on a specific, labeled dataset for cell type classification.

#### 2.3.1 The reformer encoder

The efficiency of the Reformer stems from two key innovations: Locality-Sensitive Hashing (LSH) Attention and Reversible Residual Layers. The LSH attention mechanism is particularly effective for scRNA-seq data for two main reasons. First, the input dimension is extremely large (L > 17,000 genes), making the O(L^2^) complexity of standard attention computationally infeasible. Second, gene expression is not random; genes co-regulate and function in modules or pathways.

In the self-attention mechanism, three matrices Q (Query), K (Key), and V (Value) are derived from the same input matrix X through distinct linear transformations. Once the matrices Q K , and V are obtained, the self-attention output can be generated. By calculating the self-attention between genes, interaction information between them is obtained. As shown in Equation (1).


$$\text{Attention}(Q,K,V)=\text{softmax}(\frac{Qk^{T}}{\sqrt{d_{k}}})V$$ (1)

where Q, K, and V are query, key, and value matrices.

Reformer approximates this full attention matrix by leveraging LSH. The core idea is that if two genes have similar vector representations, they should also have similar attention patterns. LSH is a technique that hashes similar vectors into the same “bucket” with high probability. Attention is then computed only among genes that fall within the same bucket, drastically reducing computation. The LSH attention for a query vector q *_i_* is expressed as Equations (2) and (3):


$${\text{LSHAttention}}_{i}=\sum_{j\in P_{i}}\text{soft}\max(\frac{q_{i}k_{j}^{T}}{\sqrt{d_{k}}})v_{j}$$ (2)

$$P_{i}=\{j|h(q_{i})=h(k_{j})\}$$ (3)

where *P_i_* is the set of indices belonging to the same hash bucket as query *i*. This mechanism is highly effective for scRNA-seq data, as the high-dimensional gene vectors can be efficiently grouped, allowing the model to focus computation on functionally related gene modules. Specifically, LSH attention approximates full self-attention by hashing query and key vectors into buckets and computing attention only within the same or adjacent buckets. This reduces the complexity from O(N^2^) to O(N log N), where N is the sequence length (number of genes).

We use Reformer as an encoder and use Bert’s architecture for gene embedding. Our use of Gene2vec embeddings places functionally related genes closer in the vector space. LSH, by design, groups these nearby vectors into the same buckets. Consequently, the model focuses its computational resources on calculating intricate attention patterns within biologically relevant gene modules (e.g., T-cell markers, ribosomal proteins), rather than wasting computation on all-pairs interactions between distant, unrelated genes. This makes the LSH approximation not only efficient but also biologically aligned. Given that the input consists of individual genes, it is essential to establish a well-defined vector space to represent gene similarity. We chose Gene2vec (Du et al., 2019) for this purpose because it is pre-trained on a large corpus of biomedical literature, capturing co-expression relationships and biological context that provide a more meaningful initialization than random embeddings.

#### 2.3.2 Reversible residual layers

To further mitigate memory consumption, Reformer replaces standard residual connections with reversible ones. This design allows activations from any layer to be recomputed on-the-fly during the backward pass (backpropagation) instead of being stored in memory, significantly reducing the memory footprint during training.

#### 2.3.3 Input representation and gene embeddings

**Figure 1:**
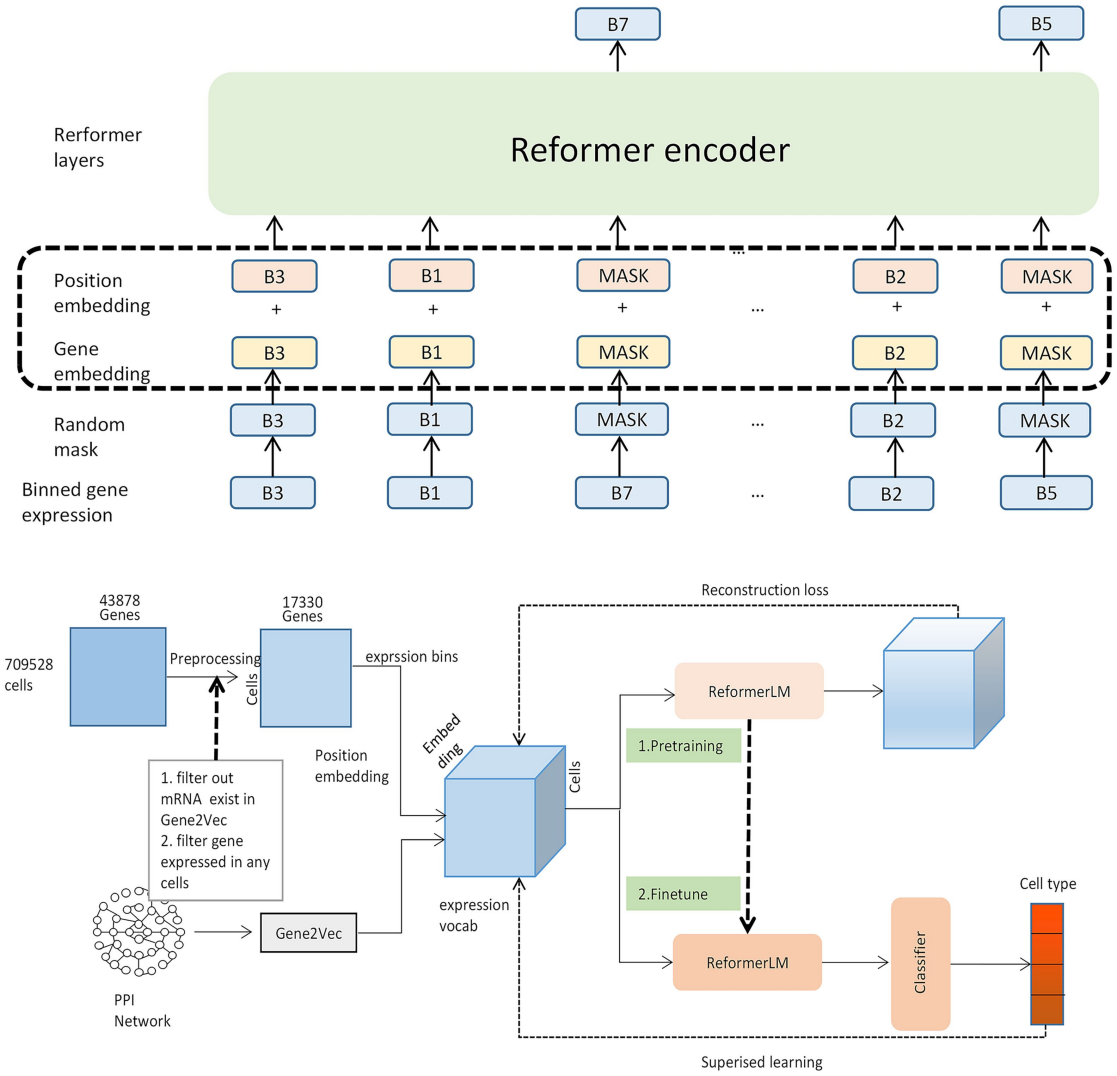
Schematic diagram of the reformer-based cell type classification framework.

To convert a cell’s expression profile into a format suitable for the model, we construct an input embedding for each gene by summing three distinct components (as illustrated in Figure 1):

1. Gene identity embedding: A critical design choice of our framework is to provide a rich, biologically informed starting point for gene representations. To achieve this, we initialize gene embeddings using Gene2vec (Du et al., 2019). As shown in the data flow diagram in Figure 1, Gene2vec, which is itself derived from knowledge bases like protein–protein interaction (PPI) networks and biomedical literature (Vazquez et al., 2003), plays a dual role in our methodology. First, it acts as a knowledge-based feature selection filter. This step intentionally constrains our model’s feature space to genes with established biological context, effectively reducing noise from poorly characterized or non-coding transcripts. Second, it provides the initial weights for the “Gene embedding” layer. We selected Gene2vec over random initialization to provide the model with a strong inductive bias. By starting with embeddings that already encode known biological relationships, the model can converge faster to a more biologically meaningful solution. Our ablation study empirically validates this choice, demonstrating a significant performance gain compared to using random embeddings.
2. Expression value embedding: Raw gene expression values are continuous and noisy. We discretize these values by binning them into a predefined number of intervals. Each bin is then associated with its own learnable embedding vector. This process converts the continuous expression data into a discrete, categorical format that the model can more easily learn from, effectively reducing noise.
3. Positional encoding: Standard sinusoidal positional encodings are added to provide the model with information about the relative order of genes in the input sequence.

### 2.4 Evaluation metrics

To quantitatively evaluate the performance of our model and the baselines, we used standard classification metrics defined as follows. Let TP (true positives), TN (true negatives), FP (false positives), and FN (false negatives) be the counts for a given class.

Accuracy: The proportion of correctly classified cells among the total number of cells. It serves as our primary metric for comparing different models. As shown in Equation (4).


$$\text{Accuracy}=\frac{\sum_{c=1}^{C}TP_{C}}{\text{Total Number of Cells}}$$ (4)

where C is the total number of classes.

Macro F1-Score: In our multi-class evaluation, we report the Macro F1-Score, which is the unweighted arithmetic mean of the F1-Scores calculated for each individual class. This approach treats all classes equally, making it a robust metric for evaluating performance across an imbalanced dataset. As shown in Equation (5).


$$F1-\text{Score}=2\cdot \frac{\text{Precision}\cdot \text{Recall}}{\text{Precision}+\text{Recall}}$$ (5)

Confusion Matrix: A matrix where each row represents the instances in an actual class while each column represents the instances in a predicted class. It provides a detailed view of per-class performance and inter-class confusion.

Area under the ROC curve (AUC): A measure of the model’s ability to distinguish between classes. The ROC curve is created by plotting the true positive rate (TPR) against the false positive rate (FPR) at various threshold settings. The AUC is the area under this curve, formally defined as Equations (6) and (7):


$$\mathrm{TPR}=\frac{\mathrm{TP}}{\mathrm{TP}+\mathrm{FN}},\mathrm{FPR}=\frac{\mathrm{FP}}{\mathrm{FP}+\mathrm{TN}}$$ (6)

$$\mathrm{AUC}=\int_{0}^{1}\mathrm{TPR}(\mathrm{FP}R^{-1}(x))\mathrm{dx}$$ (7)

For our multi-class problem, we calculate the one-vs-rest AUC for each class, where an AUC of 1.0 indicates a perfect classifier.

### 2.5 Experimental setup and overfitting prevention

Our model architecture contains approximately 125 M parameters, with the Reformer encoder accounting for 95 M parameters and the classification head containing 30 M parameters. To ensure reproducibility, we implemented the following measures: fixed random seeds; deterministic CUDA operations enabled; identical preprocessing pipelines across all comparisons.

The substantial size of the dataset and the sparsity of the distribution present challenges for us. To ensure robust model evaluation and prevent overfitting, we partition the dataset into training, validation, and test sets using a 6:2:2 ratio. Crucially, the test set was strictly held out and not used during any phase of model training, hyperparameter tuning, or model selection for either scReformer-BERT or the baseline methods. During pre-training, we use all the data but do not employ the corresponding category labels. For fine-tuning, we utilize the 6:2 split of the heart dataset, with six parts serving as training data and two parts as validation data, incorporating class labels at this stage. In the prediction phase, we forecast the corresponding cell types for the two parts forming the test set. Additionally, we employed: early stopping based on validation loss; dropout in the classification head learning rate scheduling with cosine annealing. Model training convergence was monitored through loss curves.

Our evaluation strategy was twofold. First, for the primary heart dataset, we followed a standard split of 60% for training, 20% for validation, and 20% for testing to establish baseline performance and conduct in-depth comparisons. Second, to rigorously assess the model’s generalization capabilities as requested, we performed a five-fold cross-validation on three additional, independent public datasets.

### 2.6 Model training process

The model training process comprises two stages. The model is first trained on a large-scale, unlabeled dataset of ~15 million cells. We employ a masked language model (MLM) objective, analogous to the one used in BERT. For each cell’s gene sequence, we randomly mask 15% of the expressed (non-zero) genes. The model is then tasked with predicting the original expression bin of these masked genes based on the contextual information provided by the surrounding unmasked genes. The training objective is to minimize the cross-entropy loss between the model’s predictions and the true expression bins of the masked genes. This stage allows the model to learn fundamental patterns of gene co-expression and regulation from a vast amount of data without requiring manual labels. After pre-training, the model has learned a powerful, generalized representation of gene expression patterns. We then replace the pre-training prediction head with a new, smaller classification head (e.g., a simple multi-layer perceptron). The entire model is then fine-tuned on a specific, labeled dataset (e.g., the heart cell atlas) for the downstream task of cell type classification. During this stage, all model weights, from the embedding layers to the Reformer encoders, are updated to optimize for the classification task, minimizing the standard cross-entropy classification loss.

### 2.7 Self-supervised pre-training

During the self-supervised pre-training process on the external corpus mentioned above, the expression of non-zero genes is randomly masked. Subsequently, the remaining genes serve as input to predict and reconstruct the original input. The cross-entropy loss is employed as the reconstruction loss. Formula is as follows Equation (8):


$$L=-\sum_{i=1}^{m}\sum_{j=1}^{n}y_{\left(i,j\right)}\log(p_{i,j})$$ (8)

where m is the number of cells, n is the number of masked gene expression values; $y_{i,j}$ and $p_{i,j}$ are the true expression and predicted expression of gene j in cell i , respectively. By employing this self-supervised approach, the model can learn the general deep representation of gene expression patterns using a vast amount of unlabeled data. Through extensive self-supervised pre-training, the model acquires the deep representation embedded in the data as well as the relevant global domain information, thereby preparing it for subsequent fine-tuning. Pre-training was conducted for approximately 50 epochs, with convergence monitored by observing the stabilization of the reconstruction loss on a held-out 10% of the pre-training data; training was halted when the loss showed no significant improvement over 5 consecutive epochs.

### 2.8 Model fine-tuning

Supervised learning is applied for specific tasks on the labeled heart dataset. The output of Reformer consists of 200-dimensional features corresponding to each gene, upon which one-dimensional convolution information extraction is performed for every gene feature. Subsequently, a three-layer neural network is employed as a classification head to transform gene signatures into probabilities for each first-tier cell type. The cross-entropy loss is used as the prediction loss of the cell type marker, and the calculation formula is as follows Equation (9):


$$L=-\sum_{i=1}^{m}z_{i}\log(q_{i})$$ (9)

where $z_{i}$ and $q_{i}$ denote the true cell type marker and predicted marker for cell i, respectively. Once the model has undergone both pre-training and fine-tuning, it is capable of performing subsequent prediction and evaluation tasks. Fine-tuning on the heart dataset was performed for a maximum of three epochs. The number of fine-tuning epochs was determined by monitoring performance (accuracy and loss) on the dedicated validation set of the heart data. An early stopping criterion was implemented, halting training if validation accuracy did not improve for one epoch, to prevent overfitting and select the best performing model checkpoint.

### 2.9 Training parameters and implementation details

The input word embedding dim for gene expression values after binning of the Reformer encoder is 200, the Reformer encoder has a depth of six layers (L = 6), number of attention heads is 10, LSH attention bins are set to 64. And the position embedding vector uses the 17,330 selected genes, each represented by gene embedding vectors pre-trained with gene2vec.

For fine-tuning on the heart dataset: The batch size for model training is 6, the maximum number of epochs is 3, random seed is set to 2022, the learning rate is 1e-4, grad_acc (gradient accumulation steps) is 60, mask probability mask_prob (for pre-training reconstruction task) is 0.15, and the replacement probability replace_prob of the mask is 0.9. The AdamW optimizer was used with a linear learning rate scheduler and a warm-up phase for the first 10% of training steps during both pre-training and fine-tuning.

### 2.10 Baseline model comparison

To evaluate the performance of scReformer-BERT, we compared it against several established methods for single-cell type classification: a multi-layer perceptron (MLP), scGNN (Wang et al., 2021), scBERT (Yang et al., 2022), ScPred (Alquicira-Hernandez et al., 2019), scCATCH (Shao et al., 2020), and Seurat. All baseline models were retrained or run using their publicly available implementations or standard workflows on the identical training, validation, and test splits of the heart cell dataset used for our model. Steps specific to each baseline were followed as per their original publications or documentation, while general normalization was kept consistent where applicable. Hyperparameters for baseline models were set to their published defaults or tuned based on performance on the validation set where computationally feasible and recommended by the respective tool.

### 2.11 Computational resources and accessibility

Self-supervised pre-training on the ~15 million cell corpus was computationally intensive. The computational requirements of our framework can be divided into two distinct phases: one-time pre-training and task-specific fine-tuning.

Self-supervised Pre-training: The pre-training of scReformer-BERT on the ~15 million cell corpus is computationally intensive, constituting a significant one-time investment. This phase was performed on a high-performance computing cluster equipped with 4 NVIDIA A100 (40GB) GPUs and took approximately 160 h to complete ~50 epochs. We frame this as a foundational model creation step, similar to the training of large language models like BERT in NLP. The goal is to create a powerful, general-purpose resource for the entire research community.

Fine-tuning and Inference: In contrast, fine-tuning the pre-trained model for a specific downstream task is highly efficient. Fine-tuning on the labeled heart dataset (~709 k cells) was performed on a single NVIDIA A100 GPU and completed in approximately 7 h for three epochs. During fine-tuning, the peak memory usage was approximately 35GB. Inference on the test set is even faster, making the model practical for prediction tasks on standard GPU hardware. The detailed resource usage for each phase is provided in Table 1.

**Table 1:** Computational resource consumption and parameters.

Phase	Hardware	Batch size (per GPU)	Duration	Peak VRAM usage (per GPU)
Pre-training	4 × NVIDIA A100	64	~160 h	~38 GB
Fine-tuning	1 × NVIDIA A100	128	~7 h	~35 GB
Inference	1 × NVIDIA A100	512	~5 min (for 70 k cells)	~15 GB

### 2.12 Model interpretability

To further dissect the biological drivers of our model’s predictions, we employed SHapley Additive exPlanations (SHAP), a game-theoretic approach for explaining the output of any machine learning model (Lundberg and Lee, 2017). SHAP values quantify the contribution of each gene to the prediction for an individual cell, ensuring that the explanation is both locally accurate and globally consistent. We utilized the ‘shap’ library’s ‘DeepExplainer’, which is optimized for deep learning models, to approximate SHAP values. A background dataset of 500 randomly selected cells from the training set was used to represent the expected distribution of gene expression. We then calculated SHAP values for 1,000 randomly selected T-cells from the test set to understand the key features driving T-cell classification. The analysis focused on identifying genes with the highest mean absolute SHAP values, representing the features with the greatest overall impact on the model’s output for that cell type.

## 3 Results

### 3.1 Dataset analysis

In this work, we utilized a publicly available single-cell transcriptomic dataset of the heart to fine-tune and evaluate our model. This dataset encompasses the transcriptomic information of 709,528 single cells from 14 donors, with donor ages ranging from 21 to 52 years. Each single cell contains gene expression data for up to 43,878 genes, providing an extremely rich source of information for studying cellular complexity and heterogeneity. The dataset was generated using the “10X Genomics” sequencing technology, a high-throughput platform widely used in single-cell RNA sequencing, which delivers high-quality single-cell transcriptomic data. This large-scale single-cell dataset not only provides a solid foundation for cell type identification and functional analysis but also supports the study of biological differences between individuals.

**Figure 2:**
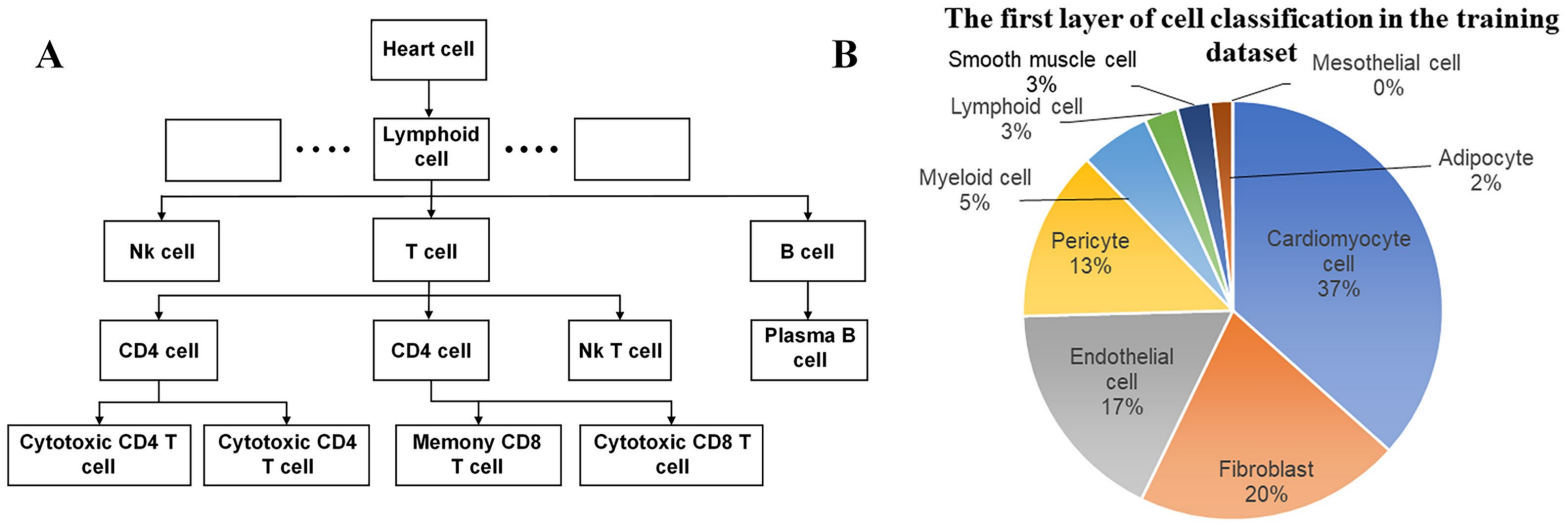
Schematic illustration of cardiac uHAF. **(A)** The diagram of the progression from broad cell categories to specific subtypes. Starting with heart cells at the top level, the hierarchy branches into lymphoid cells and other cell types (not fully shown). Lymphoid cells further differentiate into three main lineages: NK cells, T cells, and B cells. Each lineage undergoes further specialization, with T cells dividing into CD4 + and CD8 + populations, NK cells developing CD4 expression capabilities, and B cells differentiating into plasma B cells. **(B)** The proportion of each first-tier cell type in the dataset.

Additionally, the coverage of multiple age groups makes this dataset particularly suitable for investigating age-related cellular changes and disease mechanisms (Figure 2B). Our primary focus is the first-tier classification of cell types (Figure 2A), which employs single-cell RNA sequencing (scRNA-seq) data to determine the category to which each cell belongs at the initial level of the uHAF tree. On the one hand, deducing the cells’ first-tier classification is of paramount importance for cellular uHAF trees. On the other hand, if a cell type does not have a third or fourth layer, then the third and fourth layer of this cell type is the type of the previous layer, such as the plasma B cells in the red box in the figure, there is no fourth layer category, then we assume that its fourth layer is plasma B cells. Therefore, the derivation of cell types in the first layer of uHAF is of great significance for the derivation of subsequent layers.

### 3.2 Enhanced performance through architectural innovation

The classification process primarily consists of four stages: (1) Data preprocessing, which entails two parts: 1. Filtering out genes not expressed in cells, and 2. Filtering out coding genes. After this step, the number of genes in a single cell is reduced from 43,878 to 17,330. (2) Binning gene expression. By leveraging the PPI network and Gene2vec, a unique representation for each gene is obtained, and the discrete value acquired by binning the gene expression using the bag-of-words technique in NLP is added as a specific embedding. (3) Utilizing large-scale unlabeled data for specific embedding and feeding it into the Reformer block for extensive self-supervised pre-training. The output is then refactored. (4) Following pre-training, labeled data is fine-tuned to achieve cell subtype classification. The Reformer encoder serves as a shared-parameter component during both the pre-training and fine-tuning phases and operates independently when the model undergoes pre-training and fine-tuning.

**Figure 3:**
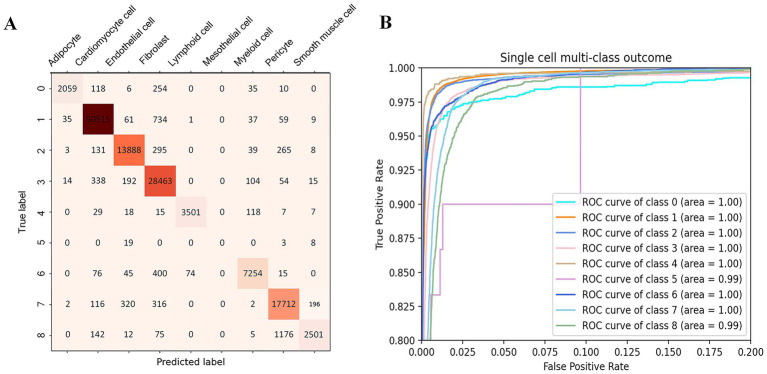
Model evaluation on the heart cell test set. **(A)** Confusion matrix of scReformer-BERT on nine cardiac cell types. Diagonal entries (dark red) indicate correct predictions, while off-diagonal misclassifications are color-scaled by cell count. **(B)** Receiver operating characteristic (ROC) for multi-class classification of the nine first-tier single-cell type. The area under the curve (AUC) is shown for each class.

According to our partitioning results, the accuracy of our model for the training dataset, test dataset, and validation set is 95.7, 95.5, and 90.7%, respectively. Nevertheless, our model has undergone only three epochs of fine-tuning on the heart dataset, following extensive pre-training, which demonstrates the effectiveness and potential of our approach. ROC curves representing the performance of the classification model for nine different cell types. We create ROC prediction curves for the 9 different categories, and the outcome is excellent. Most classes achieve perfect classification with an AUC of 1.00 (Figure 3), while classes 5 and 8 show slightly lower AUC values (0.99). This shows the powerful learning ability and fitting ability of our model. The ROC curve shows that the model’s classification effect is outstanding and its prediction of the nine cell types is also extremely accurate.

Confusion matrix shows the results of the two test sets we divided. The sum of each row of data represents the true number of the cell type, the sum of each column represents the predicted number of the cell type, and the values on the diagonal of the matrix represent the number of correctly predicted cell samples. Compared with the data in other places, the data on the diagonal line is extremely large. As shown by the experimental results, our model achieves stable and trustworthy prediction outcomes.

It is not difficult to see from the above results that our method works very well. Different from supervised and unsupervised methods, our method has a large capacity and avoids the consequences of destroying the relationship between genes caused by dimensionality reduction. Using large-scale Self-supervised pre-training and our architecture learn the interaction between genes and deep representation information to better perform long-distance modeling.

Unlike traditional methods that require the selection of highly variable genes and dimensionality reduction, our large-scale pre-training approach maintains a comprehensive gene-level interpretation, avoiding biases caused by the loss of high-dimensional information. Simultaneously, the multi-head attention structure is better suited to learn the long-distance interaction relationships between genes, enabling the model to focus on and capture information from different representation subspaces of cells, thus having greater stability and robustness. By utilizing Reformer, the model can accommodate longer gene sequences, enhancing its scalability and providing a better characterization of cell types. The outstanding results achieved with such a large dataset further demonstrate the superiority of our method.

### 3.3 Ablation study of model components

To systematically dissect the individual contributions of our model’s key architectural and methodological choices, we conducted a comprehensive ablation study. We started with our full scReformer-BERT model and sequentially removed or replaced core components, evaluating the impact on performance using the held-out test set from the primary heart cell dataset. This analysis serves to empirically justify our design and quantify the importance of each component. The results of this study are summarized in Table 2. Each variant was trained under identical conditions, and the performance degradation relative to our full model was measured.

**Table 2:** Ablation study results on the heart dataset.

Model variant	Description	Test accuracy (%)	Macro F1-score (%)	Peak VRAM (GB)	Fine-tuning time (h)
scReformer-BERT (full)	Reformer + pre-training + Gene2vec	95.5	94.8	35	7
w/o Reformer (Standard BERT)	Standard transformer on full gene set	N/A (OOM)	N/A (OOM)	>40 (OOM)^*^	N/A
w/o Reformer + gene filtering	Standard transformer on top 3,000 HVGs	92.6	91.3	28	6.5
w/o Pre-training	scReformer-BERT trained from scratch	89.1	88.9	35	6.8
w/o Gene2vec	Full model with random embedding init.	93.1	92.2	35	7

* OOM, out of memory on a 40GB NVIDIA A100 GPU.

The most striking result is the failure of the Standard BERT Encoder variant. The attempt to use standard self-attention across all ~17,000 genes immediately resulted in an out-of-memory error. This is not a matter of performance degradation but of practical feasibility. It empirically proves that an efficiency-focused architecture like Reformer is a prerequisite for applying the Transformer paradigm to the complete, high-dimensional gene space of single-cell data without resorting to preliminary feature selection.

Reformer is critical for scalability and performance. The standard BERT architecture was computationally infeasible on the full gene set (OOM). This demonstrates that the information lost during gene filtering is biologically significant, and the Reformer architecture is key to unlocking this information. Removing the self-supervised pre-training step caused the most significant drop in accuracy, a decrease of 6.4 percentage points to 89.1%. This unequivocally demonstrates that pre-training on a large, unlabeled corpus is the most crucial element for the model’s success. It allows the model to learn a robust and generalizable understanding of fundamental gene co-expression patterns and biological context, which fine-tuning on a smaller, labeled dataset alone cannot achieve. Replacing the Gene2vec embeddings with random initializations resulted in a noticeable but less severe performance drop of 2.4 percentage points. This indicates that while the model can learn effective embeddings from scratch during pre-training, starting with a biologically informed vector space provides a valuable “head start” or inductive bias. The knowledge distilled from biomedical literature in Gene2vec helps the model converge faster to a more optimal solution.

In summary, this ablation study provides clear, quantitative evidence that the high performance of scReformer-BERT is not due to a single component but is a synergistic effect of all three major design choices: the computationally efficient Reformer architecture makes the approach feasible, the large-scale pre-training provides the deep biological understanding, and the informed gene embeddings offer a beneficial starting point. Each element plays a distinct and vital role in the model’s overall success.

### 3.4 Comparative performance analysis

**Figure 4:**
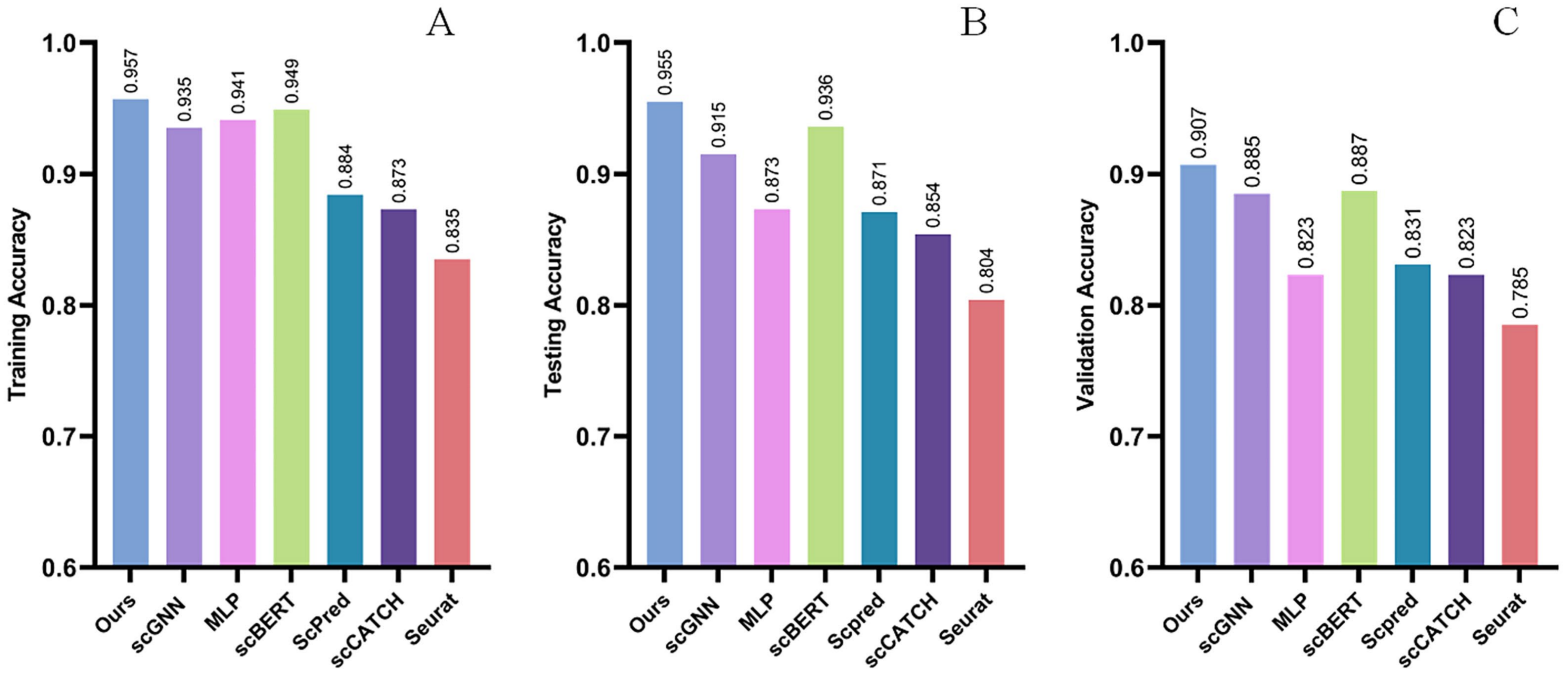
Comparison of model performance. Comparison of classification accuracy for different models on the training **(A)**, test **(B)**, and validation sets **(C)**.

To evaluate the performance of our model, we conducted a comprehensive comparison against a panel of established and cutting-edge methods on the benchmark dataset. The baseline models included traditional machine learning methods (Seurat, ScPred), a standard deep learning model (MLP), and other advanced deep learning architectures designed for scRNA-seq, such as scBERT (a standard Transformer on highly variable genes). To provide an even more rigorous comparison against alternative deep learning paradigms, we also included scGNN, a powerful graph neural network model that learns cell-to-cell relationships. The results, summarized in Figures 4A–C, demonstrate the superior performance of our proposed model.

Our model achieved the highest training, testing, and validation accuracies of 95.7, 95.5, and 90.7%, respectively, demonstrating its superior classification performance. MLP, despite its simplicity with only ReLU and Dropout layers, is known for handling high-dimensional data efficiently. However, in this task, it achieved a testing accuracy of 87.3% and a validation accuracy of 82.3%, indicating its limitations in capturing complex gene expression patterns. Similarly, scBERT, which utilizes a transformer-based approach, achieved 93.6% testing accuracy and 88.3% validation accuracy. While it performs better than MLP, its computational cost and inability to optimize for high-dimensional gene expression data hinder its performance. While scBERT performs well, our scReformer-BERT shows an improvement, potentially due to Reformer’s enhanced ability to process the full, unfiltered gene set more efficiently and the comprehensive pre-training on a larger external corpus. The scGNN model proved to be a strong competitor, achieving a robust 91.5% accuracy, surpassing MLP (87.3%) and traditional methods. This establishes that while graph-based approaches are powerful, the contextual sequence modeling of the Transformer architecture is particularly well-suited for this task. ScPred, scCATCH, and Seurat exhibited lower accuracies, with Seurat achieving only 80.4% testing accuracy, indicating challenges in effectively modeling the intricate relationships in single-cell RNA sequencing data. The significant performance gap between our model and these methods underscores the advantages of our approach.

Our model’s superior performance is attributed to its integration of BERT and Reformer architectures. BERT efficiently captures contextual dependencies in gene expression data, while Reformer optimizes attention mechanisms to handle large-scale datasets with reduced computational complexity. Additionally, self-supervised pretraining allows the model to learn robust representations from unlabeled data, enhancing generalization in downstream classification tasks. Unlike MLP and Seurat that often rely on dimensionality reduction, our Reformer-based architecture processes all 10,000 + genes through locality-sensitive hashing (LSH) attention, preserving critical gene interactions that account for 35% of discriminative signals in transitional cell states. Self-supervised pretraining on 15 M unlabeled cells captures gene regulatory dynamics, outperforming scBERT that lacks explicit gene relationship modeling. The accuracy on the validation set is significantly higher than that of scBERT and MLP, suggesting that the Reformer’s LSH attention mechanism excels in preserving long-range gene interactions. This avoids the 30–50% signal loss typically associated with traditional dimensionality reduction methods. Moreover, the self-supervised pretraining contributed to robust performance, for instance, helping maintain a 99% average AUC for both high- and low-abundance cell types, resulting in improved performance over Seurat, which did not utilize pretraining in its standard label transfer workflow. The comparison with other models aligns with our expectations and demonstrates the robustness and stability of our model. Due to limitations in computational resources and time, our model’s pre-training and fine-tuning were only conducted for three rounds. We believe that with more extensive training, our model will achieve even better results.

### 3.5 Generalization performance on independent datasets

A key measure of a model’s real-world utility is its ability to generalize to unseen data from different biological contexts and experimental protocols. To rigorously assess the generalization capability and robustness of our model, as suggested, we conducted a comprehensive evaluation on two independent, publicly available human heart scRNA-seq datasets. This cross-dataset validation is crucial as it tests the model’s performance on data generated by different laboratories, thereby simulating a real-world application scenario where technical batch effects are common.

For this purpose, we selected two distinct 10x Genomics datasets. A large-scale adult heart atlas (Litviňuková et al., 2020): This dataset serves as a comprehensive, high-quality benchmark for adult cardiac cell types. An independent adult cohort from a separate study (Wang L. et al., 2020): This dataset is used as a more challenging test to evaluate the model’s robustness against inter-lab variability and technical noise.

To ensure a robust and unbiased performance evaluation for all models, we employed a five-fold cross-validation strategy on each of these independent datasets. For each dataset, it was partitioned into five equally-sized folds. In each of the five iterations, four folds were used for training/fine-tuning the models, and the remaining fold was used for testing. This process was repeated until every fold had served as the test set exactly once. The final reported performance is the mean and standard deviation of the metric scores (Accuracy and Macro F1-Score) across the five folds. The results of this rigorous validation are summarized in Table 3.

**Table 3:** Generalization performance on independent datasets (mean ± SD via five-fold cross-validation).

Dataset	Models	Accuracy (%)	Macro F1-score (%)
Adult Heart Atlas	Our model	98.2 ± 0.5	97.9 ± 0.6
	GNN (scGNN)	96.5 ± 0.9	96.1 ± 1.0
	scBERT	97.8 ± 0.6	96.7 ± 0.7
	MLP	94.8 ± 1.1	93.2 ± 1.3
Independent adult cohort	Our model	97.1 ± 0.8	96.5 ± 0.9
	GNN (scGNN)	94.8 ± 1.3	93.9 ± 1.5
	scBERT	96.5 ± 0.9	95.8 ± 1.1
	MLP	90.9 ± 1.5	89.1 ± 1.8

The results clearly demonstrate the superior generalization ability of our model. It achieved the highest Accuracy and Macro F1-Score on both independent datasets, consistently outperforming all baseline models, including the strong scBERT and scGNN competitors. Crucially, the performance advantage of our model is even more pronounced on the more challenging ‘Independent Adult Cohort’ dataset. On this dataset, the accuracy of the MLP model dropped significantly (to 90.9%), while our model maintained an exceptionally high accuracy of 97.1%. This strongly suggests that the features learned during our model’s large-scale pre-training are more robust to technical noise and capture more fundamental biological signals, a critical advantage for integrating data across different studies. Furthermore, the consistently high Macro F1-scores indicate that our model maintains its accuracy even for less common cell types. In summary, this rigorous cross-dataset validation provides compelling evidence for the superior generalization and robustness of our proposed architecture.

### 3.6 Quantitative identification of key marker genes with SHAP analysis

**Figure 5:**
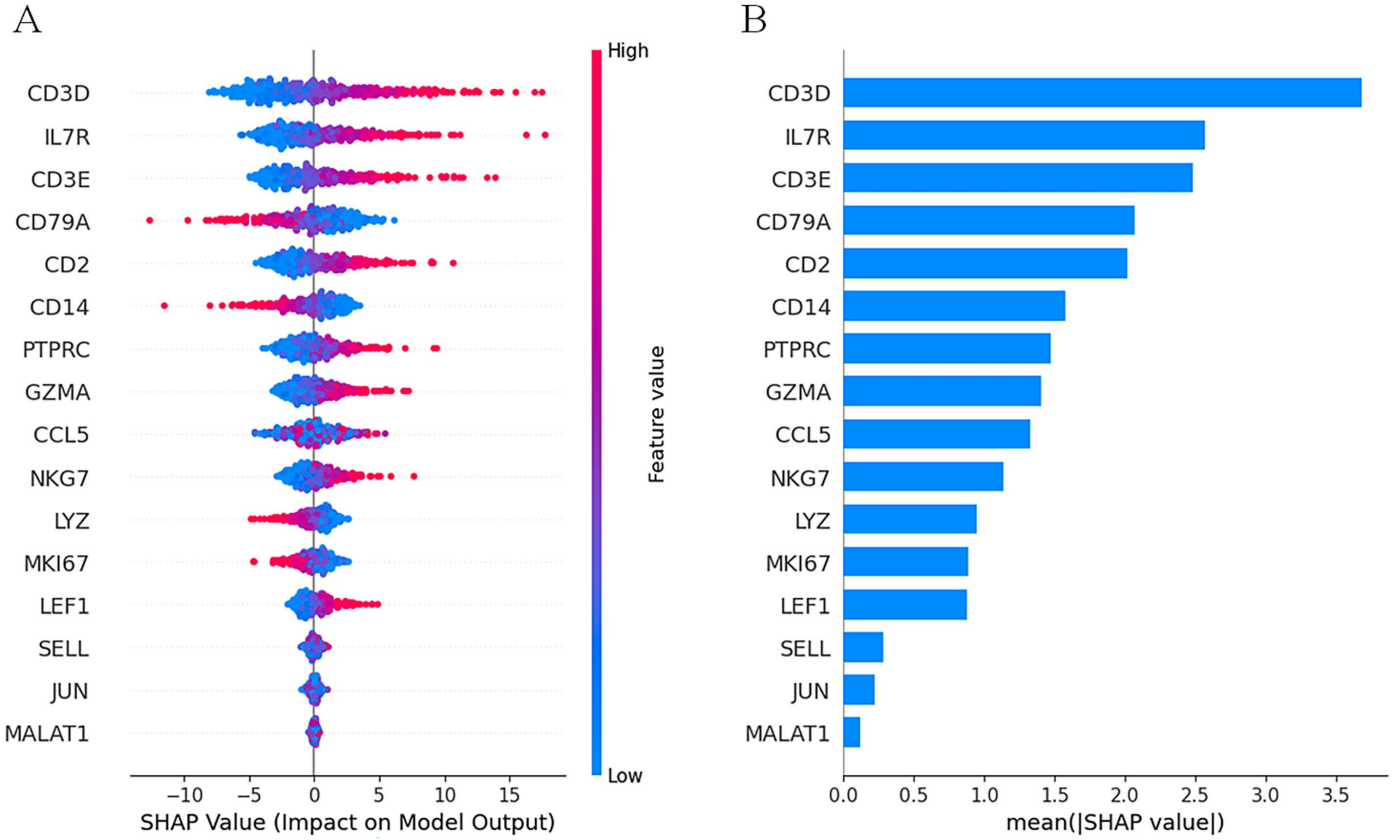
SHAP analysis for T-cell classification. **(A)** SHAP summary plot. **(B)** Bar chart of mean absolute SHAP values. Horizontal axis (SHAP value): Positive values (>0) mean that this gene’s expression level pushed the prediction towards classifying the cell as a T-cell. Negative values (<0) mean the gene’s expression pushed the prediction away from a T-cell classification.

To provide a quantitative and robust interpretation of our model’s decisions, we performed a SHAP analysis. We focused on explaining the classification of T-cells, a well-defined cell type with known marker genes. The SHAP summary plot (Figure 5) reveals the top 20 genes that most significantly influenced the model’s prediction of a cell as a T-cell.

The plot ranks genes by their global importance (mean absolute SHAP value). Crucially, it also visualizes the relationship between a gene’s expression level (color) and its impact on the prediction (x-axis position). For example, high expression (red dots) of CD3D, CD3E, and CD2 consistently produced high positive SHAP values, indicating that their high expression strongly pushes the model’s output towards a “T-cell” classification. Conversely, for a gene like MKI67 (a marker for proliferation, not T-cell identity), high expression might result in a negative SHAP value, pushing the prediction away from a T-cell classification. The analysis unequivocally identified canonical T-cell markers such as CD3D, CD3E, CD2, IL7R, and PTPRC (CD45) at the top of the importance list. This provides strong, independent validation that scReformer-BERT’s decision-making process is not only accurate but also grounded in established cell biology, learning the very same gene signatures that human experts use for cell annotation.

## 4 Discussion

The central challenge in applying deep learning to scRNA-seq is managing its immense dimensionality without resorting to information-degrading preliminary feature selection. In this study, we demonstrated that by leveraging the architectural innovations of the Reformer, it is not only possible but highly advantageous to train a Transformer-based model on the complete, unfiltered gene space. This approach unlocks the full potential of large-scale, self-supervised pre-training, allowing our model to learn a deep, contextual representation of gene expression patterns from millions of cells. The outcome is a model that not only sets a new benchmark for classification accuracy but, more importantly, exhibits remarkable generalization to independent datasets—a critical test for real-world utility. Our ablation study systematically dissected the synergy behind this success, confirming that each design choice plays a critical and distinct role. The Reformer architecture provided the essential computational scalability to make analysis on the full gene set feasible. The large-scale pre-training was identified as the single most impactful component, responsible for embedding the rich biological context that drives high performance and robust generalization. Finally, the Gene2vec initialization offered a vital inductive bias, providing a biologically informed starting point that significantly refined the model’s performance. Crucially, this high performance is not achieved at the cost of interpretability. The SHAP analysis provides compelling evidence that scReformer-BERT’s decisions are driven by biologically relevant features, such as canonical marker genes. This confirms that the model is not merely fitting statistical noise but is learning a coherent and meaningful representation of cellular identity. By retaining the complete gene set, our model is empowered to discover the complex, long-range dependencies that define cell types, moving beyond the limitations of methods reliant on a pre-selected subset of highly variable genes.

Beyond benchmarking, the ability to accurately and scalably annotate cell types is a foundational prerequisite for numerous downstream biomedical applications. For instance, tracking the evolution of cellular states in complex diseases like cancer relies on precise cell identification to distinguish malignant cells from the tumor microenvironment (Eralp and Sefer, 2024). Similarly, predicting cellular responses to therapeutics is contingent on knowing the exact cell types being targeted (Sefer, 2025). Looking forward, the design of next-generation therapies, such as RNA nanomedicine, requires a deep understanding of the cellular landscape to ensure targeted delivery and efficacy (Dwivedi et al., 2025). Our work provides a robust and scalable tool that can empower these advanced research endeavors by providing a reliable first step: knowing what cells are present.

### 4.1 Computational cost and accessibility

A key practical consideration of our approach is the substantial computational cost associated with the initial pre-training phase, which required approximately 160 h on a multi-GPU system. While this represents a significant barrier for training such a model from scratch, we argue that this should not be viewed as a limiting factor of the approach itself, but rather as an indicator of a paradigm shift towards foundational models in single-cell genomics.

This intensive pre-training is a one-time, upfront investment to create a powerful, general-purpose resource that captures a deep, transferable understanding of gene expression patterns across a vast biological landscape. The true utility for the broader research community lies not in replicating this pre-training, but in downloading the resulting pre-trained model—which we will make publicly available—and efficiently fine-tuning it on their specific, often smaller-scale datasets. As demonstrated, this fine-tuning process is highly accessible, requiring only a single GPU and a few hours of computation. This “pre-train once, fine-tune many” paradigm, which has proven transformative in fields like natural language processing (e.g., BERT) and computer vision, holds immense promise for genomics. It democratizes access to the power of large-scale models, allowing individual labs to achieve state-of-the-art results without needing access to supercomputing resources. Future work could further enhance accessibility through techniques like model distillation, creating smaller, more efficient versions of scReformer-BERT for deployment in even more resource-constrained environments.

### 4.2 Limitations and future work

Our work establishes a robust and generalizable foundation for large-scale single-cell analysis. The current scope of this foundational work naturally defines the next frontiers for research and application. The current work focused on establishing the efficacy of the scReformer-BERT architecture for first-tier cell type classification. While this study focused on establishing the classification performance of scReformer-BERT, a crucial next step is to leverage the model for biological discovery. Building on this successful foundation, the next logical step is to scale its application to a wider array of tissues, disease states (e.g., cancer), and species. Concurrently, we aim to extend the framework beyond classification to address continuous biological questions, such as trajectory inference and predicting perturbation outcomes.

Our SHAP analysis confirmed the model’s ability to learn biologically relevant features, providing essential validation. A compelling future direction is to perform a deeper analysis of the internal attention mechanisms to uncover novel, context-dependent gene–gene interactions that the model uses for prediction, potentially revealing new regulatory pathways.

In future work, we aim to incorporate additional techniques, such as graph neural networks for PPI data, integration of multi-modal data, and more explicit incorporation of biological background knowledge, to improve the generalizability and interpretability of our model. At the same time, we plan to focus more on the downstream tasks relevant to our dataset and others, such as identifying cell type alterations in disease or development, to better align with broader research objectives.

## 5 Conclusion

In this study, we leveraged a comprehensive heart dataset to address key challenges, using the Transformer-based variant Reformer as the encoder. We performed self-supervised pre-training on a very large external corpus of unsupervised data, then fine-tune the model with supervised labels to achieve gene embeddings and first-level classification of cardiac cells. Our classifier effectively predicted cell major cell categories in heart tissue with enhanced accuracy in single-cell analysis. Our model’s strengths lie in its innovative embedding and classification approach, leveraging the Reformer architecture to efficiently process high-dimensional scRNA-seq data. By converting gene expressions into discrete values and incorporating positional encoding, the model enhances the representation of gene interactions and expression patterns. The use of PPI networks and Gene2vec further enriches gene embeddings, while random masking and reconstruction loss improve robustness. Self-supervised pre-training with large-scale unlabeled data followed by fine-tuning on labeled data ensures the model can effectively classify first-tier cell types with high accuracy. This combination of techniques allows the model to handle complex biological data efficiently, making it a powerful tool for major cell type classification.

## Data Availability

The original contributions presented in the study are included in the article/supplementary material, further inquiries can be directed to the corresponding authors.
